# Analyses of Commercially Available Alcohol-Based Hand Rubs Formulated with Compliant and Non-Compliant Ethanol

**DOI:** 10.3390/ijerph18073766

**Published:** 2021-04-04

**Authors:** Timothy J. Tse, Fina B. Nelson, Martin J. T. Reaney

**Affiliations:** 1 Department of Plant Sciences, University of Saskatchewan, 51 Campus Drive, Saskatoon, SK S7N 5A8, Canada; fina.nelson@usask.ca; 2 Prairie Tide Diversified Inc., 102 Melville Street, Saskatoon, SK S7J 0R1, Canada; 3 Guangdong Saskatchewan Oilseed Joint Laboratory, Department of Food Science and Engineering, Jinan University, 601 Huangpu Avenue West, Guangzhou 510632, China

**Keywords:** alcohol, hand sanitizer, COVID-19, acetaldehyde, technical-grade ethanol

## Abstract

The COVID-19 pandemic led to panic-buying of alcohol-based hand rubs (ABHRs). In response, governmental agencies (e.g., Health Canada) permitted the sale of ABHRs formulated with “technical-grade” ethanol to alleviate the growing demand. Technical-grade ethanol contains elevated concentrations of impurities (e.g., acetaldehyde, etc.), which may exhibit dose-dependent toxicity. In this study, a rapid solvent extraction was employed to analyze gelled ABHRs via gas chromatography with flame ionization detection. In total, 26 liquid and 16 gelled ABHRs were analyzed for nine common impurities to determine compliance with Health Canada interim guidelines. Of 42 samples analyzed, 11 ABHRs appear to be non-compliant with interim Health Canada guidelines. Non-compliant ABHRs exhibited elevated concentrations of acetaldehyde, with a maximal concentration observed of 251 ± 10 µL L^−1^; 3.3× higher than currently permitted. Nonetheless, frequent testing of ABHRs should be routinely conducted to reduce the risk of consumer exposure to non-compliant ABHRs.

## 1. Introduction

Community transmission of infectious disease remains a significant concern [1], especially during the current severe acute respiratory syndrome coronavirus 2 (SARS-CoV-2; aka COVID-19) pandemic. Many municipalities have enforced stay-at-home and work-at-home orders to reduce and control community transmission of this respiratory disease. Hand hygiene is a key practice that reduces the incidence of infection [1]. Hand hygiene is best achieved by either handwashing with soap or by use of hand sanitizers. Both practices effectively reduce contaminants by removing or killing microorganisms [1]. Community education in proper hand hygiene is a key activity that can significantly reduce the spread of infectious disease through reduction of bacterial and viral counts [2]. Efficacy, followed by skin compatibility, were identified to be the most important attributes of a hand sanitizer [2].

Alcohol (e.g., ethanol) can be produced by fermentation of biomass or synthetically via direct or indirect ethylene hydration (e.g., petroleum-derived ethanol) [3,4]. However, production of synthetic ethanol often requires expensive processes, such as high energy inputs to compress ethylene and water over phosphoric acid catalysts [3,4]. Bioethanol processes are the predominant method for commercial ethanol production [5] due to their lower cost, renewability (e.g., cereal grains), and absence of toxic reagents (e.g., petroleum-based olefins). Alcohol content (e.g., 60–95%), type of alcohol (e.g., ethanol, isopropanol, or *n*-propanol), and presence of additives (e.g., H_2_O_2_) [6] are critical in deriving an alcohol-based hand rub (ABHR) product with broad spectrum utility. Typically, alcohol concentrations between 60–95% (*v*/*v*) have superior antibacterial properties [6] with higher alcohol concentrations demonstrating better activity [7]. Concentrations higher than 95% are ineffective as water is required for protein denaturation, and preparations with higher alcohol concentrations evaporate quickly. Alcohol effectively kills all enveloped viruses [6], including the novel COVID-19. Non-enveloped viruses may require higher alcohol concentrations to be effective (i.e., >70%) [6].

Alcohol-based hand rubs (ABHRs) can be available in liquid, gel, or foamable formats and are the first choice for hand hygiene in healthcare settings as they afford more effective antimicrobial activity than antiseptic soaps [8,9], improve convenience and compliance [10,11], are less time-consuming, and are less irritating than washing with soap and water [12,13,14]. Based largely on convenience ABHR in gel or foam formulations are typically favored, although liquid ABHRs typically leave less residue and dry more quickly [15]. The efficacy of ABHR is determined by the amount of hand sanitizer dispensed [16,17], the contact time [18,19], whether hands are soiled or contaminated with materials [20], and hand hygiene procedures [1,2]. ABHRs also do not eliminate all types of germs [16,17]. Therefore, washing hands with mild soap for a minimum of 20 s, is a preferred method of decontamination [21,22], as it inactivates and removes a broad spectrum of bacterial or viral particles physically from the hand.

Typically, raw materials destined for the pharmaceutical and food industry must meet specific standards for composition, such as those outlined in monographs available through the Food Chemicals Codex (FCC) and the United States Pharmacopeia (USP) [23,24]. Compliance with these standardized testing protocols ensures that materials are pure and of quality. Compliance is verified and regulated by governmental agencies such as Health Canada in Canada, and the United States Food and Drug Administration (FDA). Recently, the global transmission of COVID-19 has led to panic-buying of common necessities [25], including surface disinfectants and hand sanitizers, resulting in global shortages of both products and materials and further eroding supply and distribution chains. In response, governmental agencies (e.g., Health Canada and the USFDA) have relaxed regulations over the production of ABHRs, enabling many beverage distilleries and industrial ethanol plants to produce technical-grade alcohol to help alleviate growing demand. Furthermore, due to shortages in gelation agents, Health Canada has also eased some restrictions on substitutions for rheology modifiers [26]. This has resulted in increased availability of these alcohol-based products, from ~370 (March 2020) to ~5000 products presently (February 2021) [27]. However, many of these manufacturers possess different infrastructure and utilize different and variable feedstocks, thereby influencing the grade of the end-product alcohol and the type of impurities. Therefore, one must take extra precautions in ensuring compliance and efficacy of ABHR products which are currently available. Analyses of ABHRs, to ensure compliance and efficacy, is difficult especially if unknown impurities or materials are present (e.g., type of gelation agent in the product). Therefore, in this study, we sampled a broad variety of ABHRs purchased from local and online merchants to investigate both quality and compliance of the ethanol product. In the analyses of gelled ABHRs, we also developed a rapid method to precipitate gelation materials for downstream identification and quantification of ingredient materials using gas chromatography with flame ionization detection (GC-FID).

## 2. Materials and Methods

### 2.1. Chemicals

Alcohol standards (Table 1) are characteristic of a range of common alcohols (including fusel alcohols) produced during alcoholic fermentation and were obtained from Sigma Aldrich (Mississauga, ON, Canada). The internal and recovery standards were 3-pentanol (CAS number 584-02-1) and 4-methyl-2-pentanol (CAS number 108-11-2), purchased from Millipore Sigma (Mississauga, ON, Canada). Acetonitrile (HPLC grade), ethanol (HPLC grade), and dichloromethane (certified ACS) were obtained from Thermo Fisher (Ottawa, ON, Canada). A variety of 16 gelled and 26 liquid ABHRs were purchased locally or through online retailers between March 2020 and September 2020.

### 2.2. Extraction of Gelled ABHRs

A standard spiking solution of dichloromethane (DCM) containing the recovery standard, 4-methyl-2-pentanol (10 µL·L^−1^), was used as the extraction solvent for gelled ABHRs. Gelled ABHRs were vortexed for 20 sec with this solution at a ratio of 1:1 (sample:solvent; *w*/*v*; 1 g/mL), in 2 mL VWR microcentrifuge tubes (Mississauga, ON, Canada). The samples were then centrifuged at 10,000 rpm (Spectrafuge 24D, Labnet International Inc.; Edison, NJ, USA) for 5 min at room temperature to facilitate phase separation. The supernatant was then filtered using VWR 0.2 µm polytetrafluoroethylene (PTFE) syringe filters (Mississauga, ON, Canada) and spiked with 3-pentanol (10 µL·L^−1^), then analyzed directly via GC-FID. Liquid ABHRs were filtered with 0.2 µm PTFE syringe filters (Mississauga, ON, Canada), spiked with 4-methyl-2-pentanol (10 µL·L^−1^), and analyzed directly via GC-FID with no prior treatment. Each sample was measured in triplicate, with the exception of sample 26 (due to inadequate sample amount). Results are measured as mean ± standard deviation.

### 2.3. GC-FID Analytical Methodology

Analyses of ABHR were conducted using an Agilent 7890 GC (Santa Clara, CA, USA) equipped with a flame ionization detector (FID). Samples were injected using an Agilent 7693A (Santa Clara, CA, USA) automatic liquid sampler with a 10 µL Hamilton cemented needle syringe (Reno, NV, USA). Volatile organic compounds were separated on a 30-m Agilent J&W DB-624 Ultra Inert column (ID 0.32 mm, 1.80 µm film). The FID was operated at 250 °C with flow rates of 400 mL min^−1^ of air, 30 mL min^−1^ of hydrogen, and 25 mL·min^−1^ nitrogen. Initial oven temperature was 40 °C and held for 5 min. The temperature was increased to 225 °C at 20 °C·min^−1^ and held for 2.5 min, giving a total run-time of 16.75 min. Helium was used as the carrier gas, with a flow rate of 6 mL·min^−1^. The inlet was operated in split mode (40:1) at 140 °C and 1 µL of sample was injected. The syringe was thoroughly washed with acetonitrile between injections to avoid cross-contamination. A five-point calibration curve was derived for all the impurities listed in Table 1, achieving an R^2^ > 0.99 and an RSD ≤ 25%.

## 3. Results and Discussion

The unprecedented impact of the COVID-19 pandemic has led to a global crisis resulting in widespread panic buying of essential items [28] including hand sanitizer and surface disinfectants. This demand led to shortages of commercial essential products and resulted in government regulatory agencies temporarily permitting the use of lower quality raw materials and substitutions in ABHRs [29]. A simplified licensing approach resulted in rapid manufacturing and the availability of over 5000 hand sanitizer products [27], many of which were produced using non-USP-grade ethanol. These interim guidelines initially permitted acetaldehyde concentrations to be not more than (NMT) 1000 ppm. However, by June 2020, the concentration of permitted acetaldehyde was lowered to 400 ppm, and later reduced to 75 ppm by September 2020 [30]. This decrease in regulatory limits was attributed partly to improved distillation and purification processes at many manufacturing facilities [30]. Although, samples >75 ppm can be marketed and sold, they require additional health related labelling [30]. Typically, USP-compliant ethanol is required in the formulation of ABHRs and is limited to NMT 10 ppm of combined acetal and acetaldehyde [23]. Fortunately, many of the hand sanitizers examined in this study were compliant with Health Canada’s interim guidelines for technical-grade ethanol. However, the main non-compliant contaminant appeared to be acetaldehyde.

Typically, ABHRs are formulated using pharmaceutical-grade ethanol, examined against the USP ethanol monograph. This monograph specifically requires quantification of methanol, benzene, acetal (1,1-Diethoxyethane), and acetaldehyde in addition to a sum of all other organic compounds detected by GC. Traditionally benzene has been used as an entrainer in azeotropic distillation systems for ethanol dewatering [31], however, due to its toxicity [32] this solvent has since been replaced with other less-hazardous solvents (e.g., cyclohexane and ethylene glycol) [33]. Currently, the most established drying methods are distillation and adsorption (e.g., molecular sieve) processes [33,34]. Initial results demonstrated a compound elution at 3.90 min, which is typical of for benzene elution. This was later identified to be isobutanol and was confirmed through GC-MS. To be marketed as USP-grade ethanol, solutions must contain ≤200 µL·L^−1^ methanol, ≤2 µL·L^−1^ benzene, and ≤10 µL·L^−1^ combined acetal and acetaldehyde [23], and the sum of all other impurities must also be ≤300 µL·L^−1^ [23].

Extraction of compounds of interest from gelled ABHRs using dichloromethane was successful, achieving a mean recovery of 105.7% ± 9.1%. However, 1-butanol co-eluted with the internal standard in many of the gelled samples. Therefore, to obtain an approximate concentration for 1-butanol, the average area of the internal standard was subtracted from the total peak area at the retention time of 5 min. An ethanol blank containing 10 ppm of 3-pentanol was injected every 10 samples to compensate for instrument drift.

In total, 31 of the 42 samples complied with the interim guidelines outlined by Health Canada [30]. The remaining 11 ABHR samples (samples 4, 11, 13, 14, 15, 19, 21, 45, 25, 26, and 42) did not comply with current Health Canada legislation, primarily due to excess acetaldehyde content within the ABHRs. However, these products were purchased prior to the regulatory decrease in acetaldehyde concentrations issued by Health Canada.

**Figure 1:**
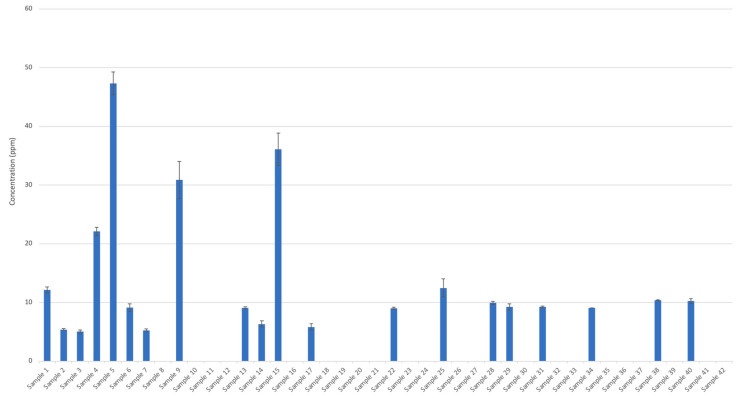
Methanol concentration in alcohol-based hand rubs (liquid, samples 1 to 26; gelled, samples 27 to 42) obtained from local and online merchants.

**Figure 2:**
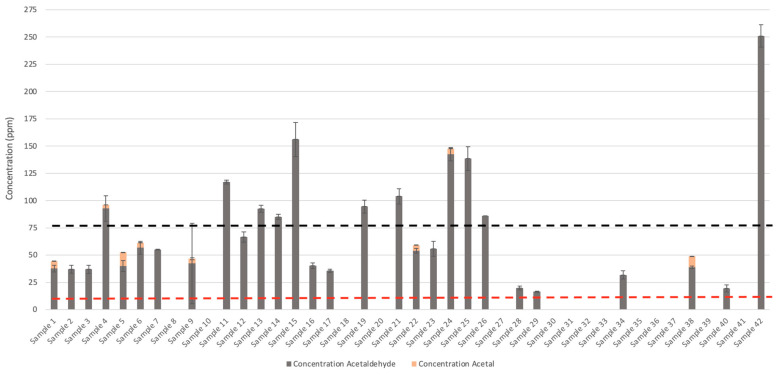
Acetaldehyde and acetal concentrations in alcohol-based hand rubs (liquid, samples 1 to 26; gelled, samples 27 to 42) obtained from local and online merchants. The grey and beige colour represent acetaldehyde and acetal, respectively. The black and red dotted line represents the interim Health Canada and United States Pharmacopeia (USP) limit, respectively, on acetaldehyde and acetal concentrations, as of September 2020.

Methanol concentrations were below 200 µL L^−1^ in accordance with USP criteria, ranging from below limited of detection (LOD) to 47 ± 2 µL L^−1^ (Figure 1). Acetaldehyde appeared to be the most common impurity in ABHRs analyzed, with 28 samples containing acetaldehyde concentrations from 17 ± 0.5 µL·L^−1^ to 251 ± 10 µL·L^−1^ (Figure 2). Acetal concentrations were between <LOD to 12 ± 0.3 µL·L^−1^, with only two samples exceeding 10 µL·L^−1^. Eleven of these samples exceeded the interim Health Canada guidelines on acetaldehyde and 17 samples exceeded the USP monograph for combined acetal and acetaldehyde. Acetaldehyde is produced through the oxidation or metabolism of ethanol by yeast and bacteria [35]. Reversible conversion of acetal to acetaldehyde can also occur in the presence of ethanol and acidic catalysts [36,37]. Therefore, for compliance of an ethanol product with USP and Health Canada requirements it is necessary to include the total acetal and acetaldehyde content to account for this conversion.

**Figure 3:**
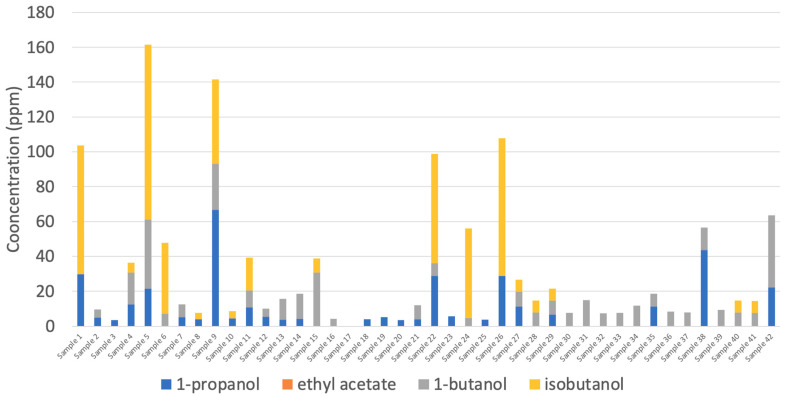
Concentration of 1-propanol, ethyl acetate, 1-butanol, and isobutanol in alcohol-based hand rubs (liquid, samples 1 to 26; gelled, samples 27 to 42) obtained from local and online merchants.

The guidelines outlined in the USP monograph and Health Canada, sum the remaining impurities together as a measure of total organic impurities. According to the USP monograph, the sum of all other impurities must not exceed 300 ppm. However, due to the COVID-19 pandemic, Health Canada temporarily increased limits for total impurities in technical-grade ethanol. Currently, if the sum of all other impurities exceeds the interim limit of 300 ppm, Health Canada requires that all individual impurities must be identified and meet the individual interim limits [30]. These other listed impurities include acetone, 1-propanol, ethyl acetate, 2-butanol, 2-methyl-1-propanol (aka isobutanol), 1-butanol, 3-methyl-1-butanol, and amyl alcohol [30]. In this study, 1-propanol, ethyl acetate, 1-butanol, and isobutanol were identified, and their concentrations were below the individual interim guidelines enforced by Health Canada (≤1000 ppm, ≤2200 ppm, ≤1000 ppm, and 21,700 ppm, respectively) (Figure 3) [30]. However, other unidentified impurities were present in some of the ABHRs sampled in this study. In the event unknown impurities are present in ethanol, Health Canada requires the manufacturer to identify these compounds and report them for assessment [30].

**Figure 4:**
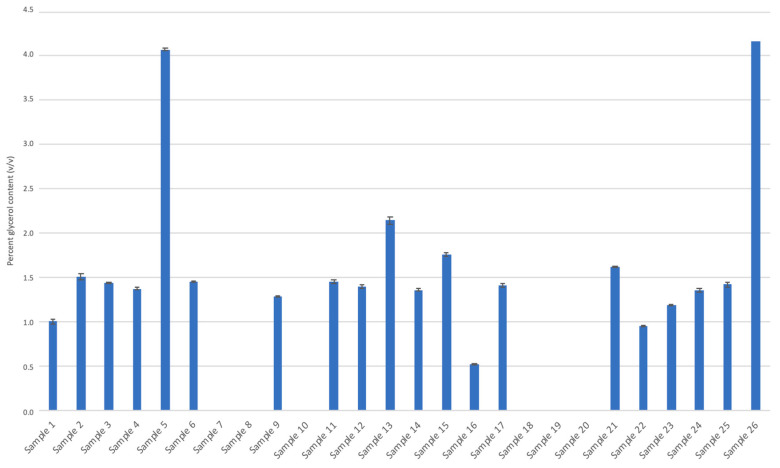
Glycerol content (%; *v*/*v*) of liquid alcohol-based hand rub samples.

Many of the ABHRs in this study exhibited the presence of humectants (e.g., glycerol and 1,2-propanediol). Humectants are typically added to ABHRs as a moisturizing agent and to encourage hand hydration [38]. In the liquid ABHRs analyzed, 20 of 26 samples contained glycerol ranging between 0.52% ± 0.01% and 4.0% ± 0.01% (*v*/*v*) (Figure 4). However, there was a single sample identified (sample 26) with a glycerol content of 4.2% (*v*/*v*). The current formulations of hand sanitizer suggested by the World Health Organization are either 80% (*v*/*v*) of ethanol or 75% (*v*/*v*) isopropanol mixed with 1.45% glycerol (*v*/*v*) [39]. Most of the liquid glycerol-containing ABHRs analyzed appeared to comply with this formula. Similarly, 1,2-propanediol was identified in a few samples. This compound is the second-most-used humectant in cosmetic products and is generally recognized as safe [38]. Similar to glycerol, this compound is general used in small concentrations, typically between 2% and 5% [38]. Nevertheless, these hand sanitizers were not labeled to indicate the presence of the 1,2-propanediol.

**Figure 5:**
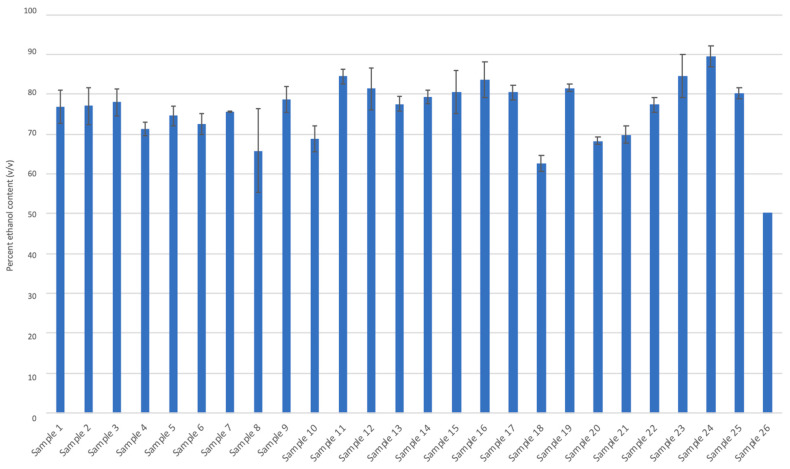
Ethanol content (%; *v*/*v*) of liquid alcohol-based hand rub samples.

Meanwhile, ethanol content was estimated by comparing the ethanol peak area in sample chromatograms against an absolute analytical-grade ethanol blank. Ethanol content in the liquid ABHRs varied between 63% ± 2% (*v*/*v*) to 90 ± 3% (*v*/*v*), although sample 26 had an ethanol content of 50% (Figure 5). With the exception of sample 26, the concentration of ethanol in these ABHRs suggested adequate viricidal activity [40,41].

Although not quantified, isopropanol was present in 12 ABHRs. Two of the gelled ABHRs contained isopropanol as the active ingredient (samples 33 and 34), while four ABHRs contained an elevated mixture of ethanol and isopropanol as the active ingredient (samples 6, 8, 18, and 29). Finally, the remaining six ABHRs (samples 4, 5, 28, 31, 32, and 42) appeared to have minimal amounts of this compound. Ethanol is the most effective alcohol against virus, whereas isopropanol is considered to be a better bactericidal alcohol [42]. However, isopropanol is fully effective against virus with lipid envelopes [40]. Therefore, the combination of these alcohols could potentially have a synergistic effect [42].

The permittance of technical-grade ethanol in ABHRs enabled small, non-traditional businesses to manufacture ABHRs to meet market demands. However, the new manufacturers implemented non-conventional packaging for ABHRs [29]. For example, one of the ABHR samples purchased was shipped in a carbonated aluminum beverage container. Interestingly, the product eventually corroded and ruptured the can after storage at room temperature for several weeks. Carbon dioxide is acidic in nature and can chemically modify ABHR composition by altering equilibrium reactions that create ethers, acetals, and esters [36,37]. This is important to note, as the USP monograph and Health Canada specifically require that acetal and acetaldehyde impurities must be summed together to account for acetal conversion to acetaldehyde. Although pH was not measured in this study, the nominal concentration of acetal could potentially be attributed to the ABHR pH, resulting in an increase in acetaldehyde concentrations.

**Figure 6:**
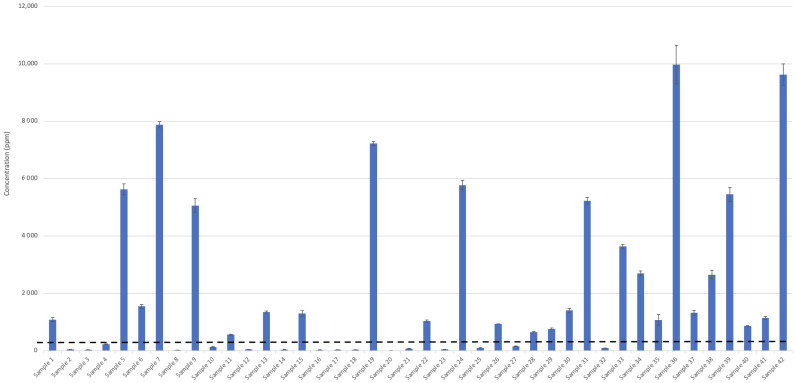
Estimated concentrations of all other impurities (summed). The dotted line represents the USP limits permitted for total impurities in alcohol-based hand rubs (liquid, samples 1 to 26; gelled, samples 27 to 42) obtained from local and online merchants.

**Figure 7:**
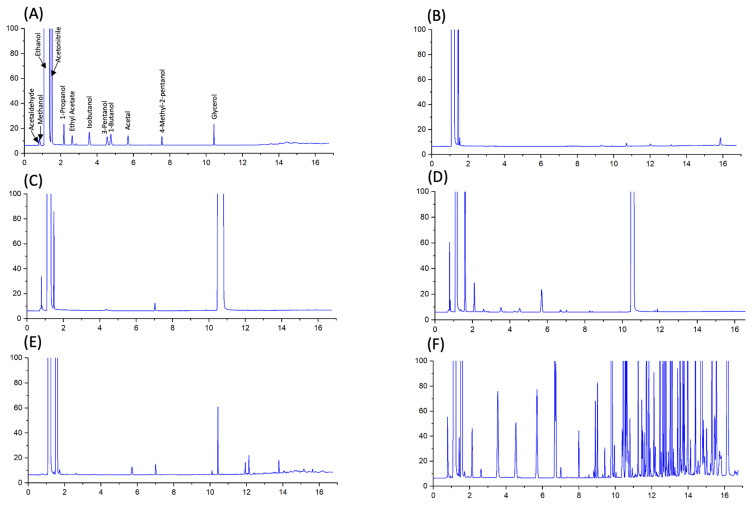
Chromatograms standard mix (**A**), USP-grade ethanol (**B**), compliant (Health Canada guideline) liquid ABHR (**C**), non-compliant (Health Canada guideline) liquid ABHR (**D**), compliant (Health Canada guideline) gelled ABHR (**E**), and non-compliant (Health Canada guideline) gelled ABHR (**F**).

Finally, analyses of ABHRs can be further complicated by the presence of additive ingredients, such as fragrances. Fragrances may be mixed into ABHR products as a possible attempt to mask odours related to fusel alcohols (e.g., sample 42; Figure 6). The addition of fragrances, or the storage container itself, can also lead to inadvertent inclusion of phthalates including diethyl phthlate. This compound is routinely used as a vehicle for fragrance and cosmetic ingredients [43] and was observed in some ABHRs sampled (via GC-MS; data not shown). However, this compound was not further investigated in this study. Other unidentified impurities were also observed in some ABHR samples, which collectively had concentrations of up to 9623 ppm ± 246 ppm (in sample 42; Figure 6), although this could be a result of additive ingredients. Chromatograms of many of the gelled ABHRs had numerous unidentified peaks (Figure 7), possibly suggesting contribution from additive ingredients (e.g., fragrances) rather than from ethanolic raw ingredient. Regardless, identification and quantification of these unknown impurities should be conducted and presented to Health Canada for review to ensure consumer safety. For most ABHRs investigated in this study, impurities that were identified and quantified suggest minimal toxicity to the consumer [44]. However, the presence of unidentified compounds can be concerning as they represent a significant portion of total impurities in several of the ABHRs analyzed. Therefore, caution should be exercised for at-risk populations. Acetaldehyde is toxic and a likely carcinogen [45] (Group 2B classification by the International Agency for Research on Cancer) and has been discovered to be the principal chemical involved in fetal alcohol spectrum disorder [46]. However, it is unlikely that acetaldehyde adsorption, at these concentrations, would reach acute toxic levels [47]; still, percutaneous toxicity in children can occur with regular application of ABHRs [47]. Nonetheless, chronic toxic effects of acetaldehyde in ABHRs have not been thoroughly investigated, and special attention should be taken into account in regard to children and individuals with genetic deficiencies in ethanol metabolism [47].

## 4. Conclusions

To conclude, because of the COVID-19 pandemic, Health Canada implemented interim guidelines to address shortages in high-quality ethanol for formulation into ABHRs and surface disinfectants. Correspondingly, there was a drastic upsurge in commercially available ABHRs, as local breweries and fuel ethanol plants gained new market entry into these sectors during the interim period. However, some manufacturers might not have capacity to refine ethanol and remove impurities. Due to the vast amount of ABHR currently on the market, vigilant testing should be routinely conducted to ensure product compliance and consumer safety. Acetaldehyde also remains a nuisance impurity that many manufacturers struggle to reduce to meet Health Canada’s interim limitations. With regard to other impurities (e.g., methanol, ethyl aetate, and 1-propanol), many of the ABHRs appear to meet Health Canada’s interim guidelines on technical-grade ethanol, with only six of the ABHRs studied meeting USP limitations on organic impurities in ethanol.

## Figures and Tables

**Table 1:** Retention times of common impurities in technical-grade ethanol.

Compound	Retention Time (min)	Limit of Detection (LOD; µL·L^−1^)	Limit of Quantification (LOQ; µL·L^−1^)
Acetaldehyde	0.86	0.15	0.46
Methanol	0.91	0.14	0.44
Ethanol	1.40	0.15	0.56
1-Propanol	2.35	0.34	1.02
Ethyl acetate	2.89	0.47	1.43
Isobutanol	3.90	0.37	0.85
1-Butanol	5.60	0.42	1.28
Acetal	6.00	0.55	1.66
Glycerol	10.70	0.13	0.39
4-Methyl-2-pentanol ^1^	7.0		
3-Pentanol ^2^	5.25		

^1^ Recover standard for gelled alcohol-based hand rubs (ABHRs) and internal standard for liquid ABHRs; ^2^ internal standard for gelled ABHRs.
